# Analysis of Quasispecies of Avain Leukosis Virus Subgroup J Using Sanger and High-throughput Sequencing

**DOI:** 10.1186/s12985-016-0559-6

**Published:** 2016-06-27

**Authors:** Fanfeng Meng, Xuan Dong, Tao Hu, Yingnan Liu, Yingjie Zhao, Yanyan Lv, Shuang Chang, Peng Zhao, Zhizhong Cui

**Affiliations:** College of Veterinary Medicine, Shandong Agricultural University, Taian, 271018 China; Institute of Pathogen Biology, Taishan Medical College, Taian, 271000 China

**Keywords:** Quasispecies-Sanger sequencing-deep sequencing-simulation

## Abstract

**Background:**

Avian leukosis viruses subgroup J (ALV-J) exists as a complex mixture of different, but closely related genomes named quasispecies subjected to continuous change according to the Principles of Darwinian evolution.

**Method:**

The present study seeks to compare conventional Sanger sequencing with deep sequencing using MiSeq platform to study quasispecies dynamics of ALV-J.

**Results:**

The accuracy and reproducibility of MiSeq sequencing was determined better than Sanger sequencing by running each experiment in duplicate. According to the mutational rate of single position and the ability to distinguish dominant quasispecies with two sequencing methods, conventional Sanger sequencing technique displayed high randomness due to few sequencing samples, while deep sequencing could reflect the composition of the quasispecies more accurately. In the mean time, the research of quasispecies via Sanger sequencing was simulated and analyzed with the aid of re-sampling strategy with replacement for 1000 times repeat from high-throughput sequencing data, which indicated that the higher antibody titer, the higher sequence entropy, the harder analyzing with the conventional Sanger sequencing, resulted in lower ratios of dominant variants.

**Conclusions:**

In sum, deep sequencing is better suited for detecting rare variants comprehensively. The simulation of Sanger sequencing that we propose here will also help to standardize quasispecies researching under different selection pressure based on next-generation sequencing data.

## Background

Avian leukosis virus subgroup J (ALV-J), the same as human immunodeficiency virus, displays extensive genetic diversity, reflecting the error prone characteristics of reverse transcriptase-dependent replication, increased recombination rate and continuous selection of more fit viral variants within fluctuating host ecosystems [1]. Therefore, a complex mixture of different, but closely related genomes named quasispecies subjected to continuous change following Principles of Darwinian Evolution such as genetic variation, competition, selection, interaction and cooperation were formed [2–7]. Viral variations, the primary cause of quasispecies phenomena, are strongly associated with quasispecies transitions, which affect the clinical manifestations of a patient and the antiviral therapeutic response [8, 9]. ALV-J was first isolated in 1988 from meat-type chickens in Britain [10], which spread throughout the world and caused significant economic losses in China over the past decades [11, 12]. ALV-J is more pathogenic and easier to mutate than other subgroups of ALV [13].

PCR-cloning-sequencing techniques, which most studies published 5 years ago have used, are recognized as golden standard in researching quasispecies [14]. Traditional research techniques could only aim at some of the variants, but analyzing all the variants was impossible. Studies of retrovirus diversity within quasispecies were benefited over the years by the development of novel sequencing technologies that extended the depth of sampling [1, 15–19]. The emergence of a new generation of high-throughput sequencing technology opened up a new access to all the haplotypes in quasispecies. Next generation sequencing increases the sensitivity significantly to identify low frequency genetic variants of HIV-1 quasispecies that may lead to high susceptibility to escape from immunity [15].

In this study, with the existing data from high-throughput sequencing, the reliability and reproducibility of MiSeq High-throughput Sequencing were investigated and compared with the conventional Sanger technique in reseaching quasispecies. The application of Sanger sequencing on quasispecies under antibody selection pressure was simulated and analyzed with bioinformatics method. Infection of ALV-J was selected as the model.

## Results

### Data filtration

In order to fully display the characterization of high-throughput sequencing, the reads appeared at least two times are retained and compared with the sequences obtained from Sanger sequencing. With the same three samples, we obtained 24 clonal sequences for Sanger sequencing and about 35000 reads per sample for high-throughput sequencing, respectively.

### Accuracy and repetition for quasispecies using Sanger and MiSeq sequencing

**Fig. 1:**
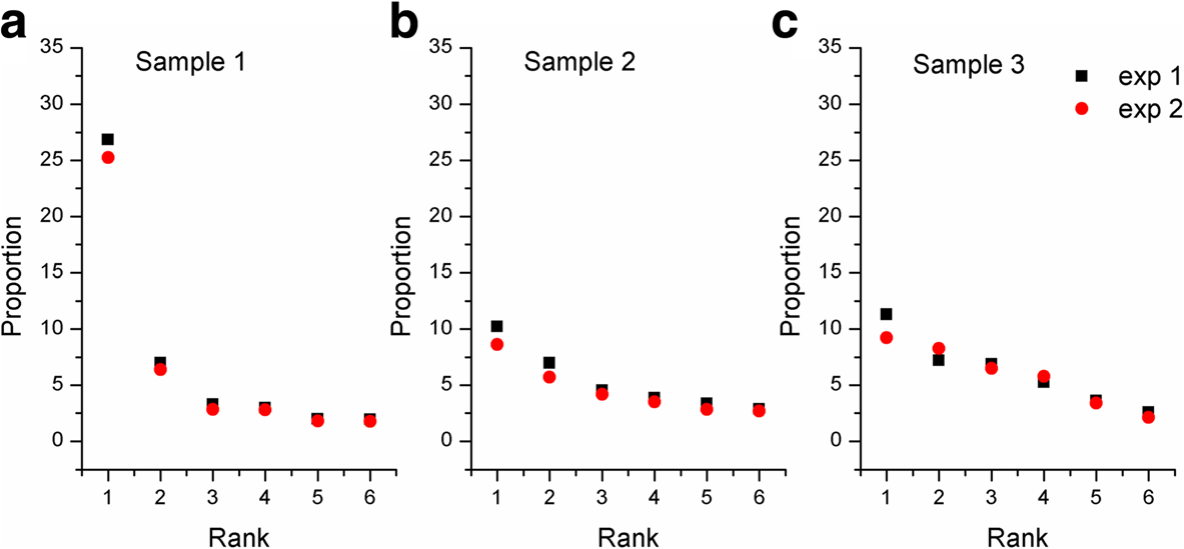
Accuracy and repetition for researching quasispecies using MiSeq sequencing

Twice Miseq sequencing were conducted with the same three different samples and found that the ranking of the top 6 variants were completely concordant. Deviation only presented slightly on the proportion of the first and second dominant variants, but did not affect the ranking of each variant (Fig. 1). Such as sample 1, the ratios of the top 3 are successively 26.86 %, 6.97 % and 3.31 % for the first round and 25.26 %, 6.38 % and 2.83 % for the second round.

In order to study the dominant quasispecies more comprehensively and accurately with the two methods, we compared the ratios of the most dominant quasispecies in segment gp85-A and gp85-B from the 3 samples at different time points. Through which, we found that there existed high randomness in distinguishing the ratios of dominant quasispecies using Sanger sequencing, when compared with MiSeq high-throughput sequencing. In other words, the proportion determined by Sanger sequencing is much higher or lower than the real ratio (Table 1).

**Table 1:** The frequency of the most dominant variant from Sanger and MiSeq Sequencing

		The frequency of the most dominant variant (%)					
Name	Methods	4w			12w		
		1	2	3	1	2	3
Segment A	Sanger	45.8	4.5	4.3	22.7	4.3	40.0
	MiSeq	26.9	10.2	11.3	10.9	9.8	13.0
Segment B	Sanger	12.5	22.7	17.4	18.2	26.0	4.0
	MiSeq	27.6	30.9	38.4	12.3	24.9	15.2

### Difference in studying mutational frequency between Sanger and MiSeq sequencing

**Fig. 2:**
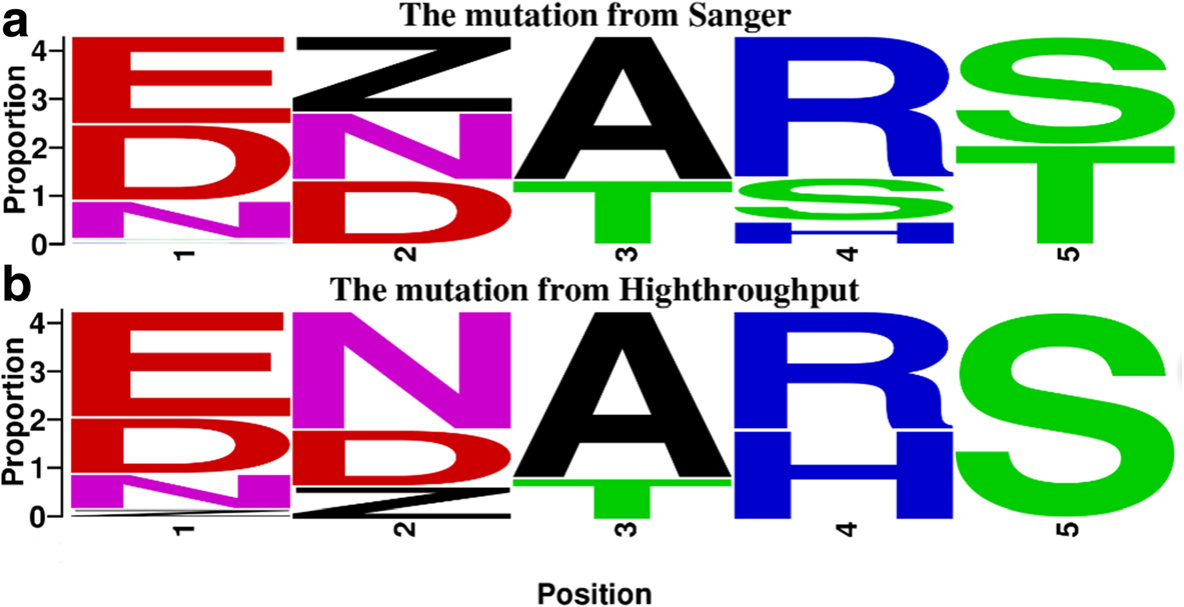
Mutational frequencies of 5 amino acids using Sanger and MiSeq high-throughput sequencing. The frequencies of 5 sites in gp85 from Sanger and Highthroughput were compared and generated by Weblogo. “Z” indicates deletion, while other capital letters indicate the normal amino acids. The size of the capital indicated the proportion of the amino acid in this position. The bigger the capital, the higher the proportion. Proportion higher than 1 % were retained for analyzing in High-throughput sequencing

Mutational rates of 5 amino acids sites got from the Sanger and high-throuput sequencing were selected and counted in gp85-A segment, we found that the mutational rates from Sanger sequencing can generally reflect the real existence condition at this position. But great randomness exists in the ratios of some specific amino acids (Fig. 2). For example, the probability of the deficiency of amino acid is 16 % ranked third at position 2 using high-throughput sequencing, but 36 % as the most dominant choice determined by the Sanger. Similar circumstances presented at position 4 and 5, where S or T amino acids accurately account for only lower than 1 % in high-throughput sequencing but 10–30 % in Sanger sequencing.

### Simulation Sanger sequencing in studying quasispecies

**Fig. 3:**
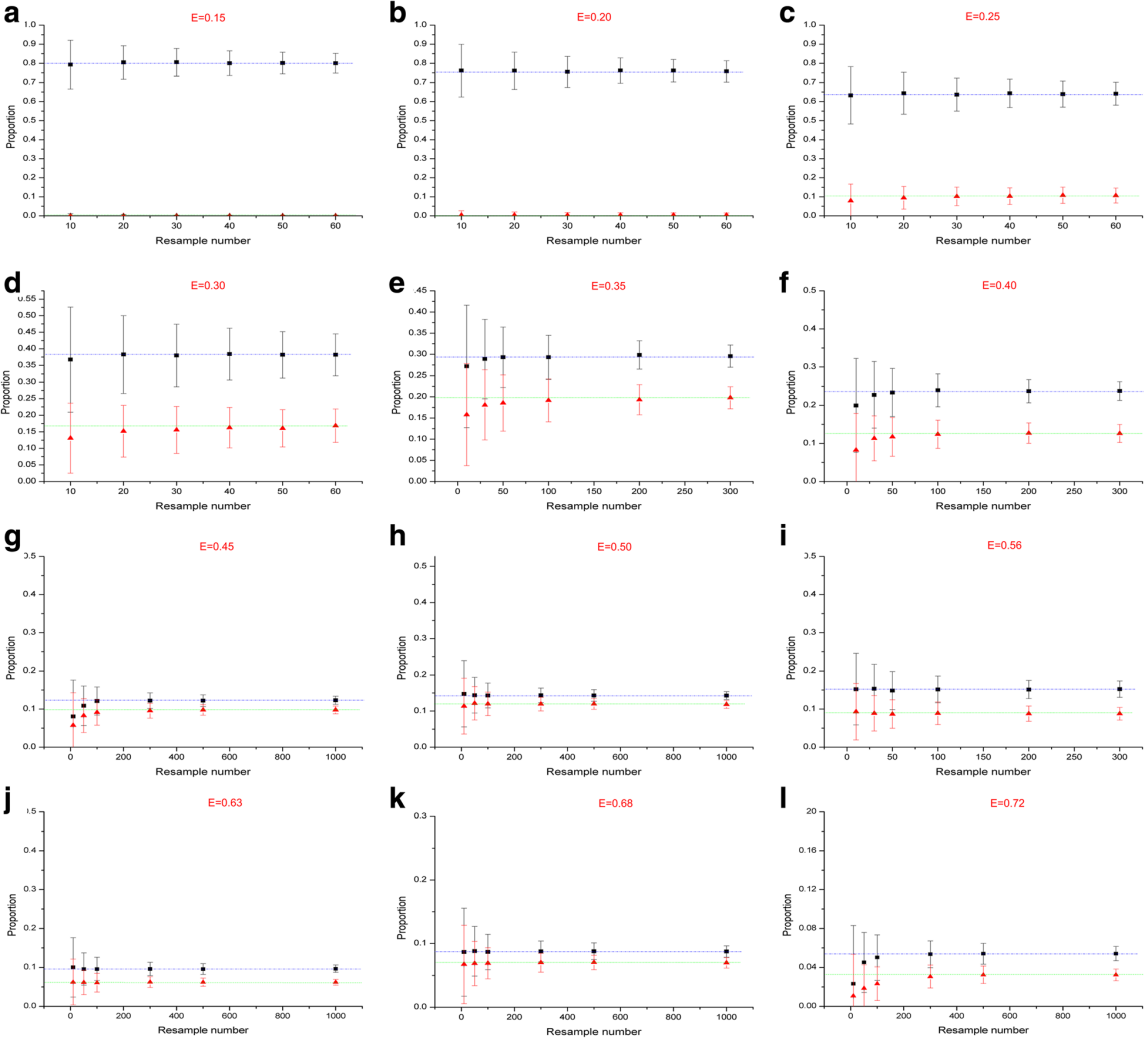
The relationship between entropy and the proportions of the first 2 dominant variants. The X axis indicates the resampling number, while Y axis represents the proportion of the top 2 variants in the n resamples. The blue dotted line indicates the true proportion of the first dominant variant; The green short dotted line represents the true value of the second dominant variant

Twelve samples with entropy ranged from 0.15 to 0.73 at different periods were collected based on the former high-throughput sequencing data. We imitated Sanger sequencing using the sampling method with replacement and then repeated. According to the results, we concluded that the number of necessary sequences to be extracted reflects the dominant quasispecies condition. Through the analysis, we found that higher entropy leads to lower sequencing similarity and more extracted sequences. With increased number of resampling, the standard deviation will be lower and more accurate. With an increase of the entropy, the proportion of the most dominant quasispecies gradually decreased from 80 % to 8 %. Moreover, the ratios of the top two dominant quasispecies grew closer (Fig. 3).

### Relationship between entropy and antibody value

**Fig. 4:**
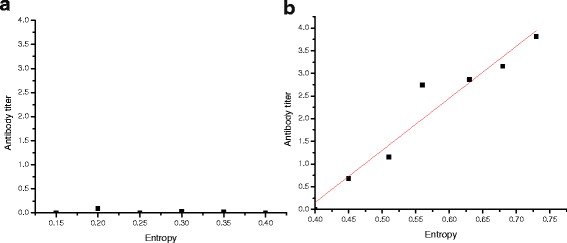
The relationship between antibody of ALV-J and sequence entropy. (**a**) The relation between samples with negative ALV-J antibody and sequence entropy; (**b**) The linearity between samples with different values of positive ALV-J antibody and sequence entropy

Entropy value of the three consistent viremia with positive antibody chickens at different time points was calculated. The max entropy was 0.44 for chickens with negative antibody. But there is no regular pattern when the antibody values lower than 0.4, which may be caused by the individual difference (Fig. 4a). The antibody value higher than 0.45 had linear relationship with entropy, namely the value of entropy will present linear growth with the increase of ALV-J antibody (Fig. 4b). A formulas *y* = 10.949×-4.096 via linear statistical analysis was got. The higher the antibody value, more sequences were needed for accurately analyzing quasispecies. But the antibody value had no linear relationship with the numbers of resampling sequences, which are at least 800.

## Discussion and conclusions

Presently, there is high regard for the study of the quasispecies characteristic of RNA viruses which are related with the clinical diseases [20–22]. Quasispecies is a particular form of mutation-selection balance in which a distribution of variant genomes is ordered around the fittest, or master sequence [23]. There is very important significance in quasispecies research on the screening of virus resistant strains,viral mutants from immune escape, which let us deeply understand the interaction between virus and its external environment [24]. The evolution of viral quasispecies changed the character and the propagation mode of the retrovirus, which brought severe challenges to existing detection technology and controlling measures. Papers that researching on various diseases through different sequencing technologies have been reported [1, 19, 25]. To our knowledge,it is the first time that quasispecies of ALV-J has been studied using different sequencing methods, especially deep sequencing. The occurrence of high-throughput sequencing speed up the pace in study of the regularities and characterizations of quasispecies. Detecting low-frequency variants in the mix of PCR products via a new generation of high-throughput sequencing become more achievable and convenient. Also with the rapid development of biodata processing, the advantages of high-throughput sequencing will be further obvious.

In order to utilize high-throughput sequencing to research quasispecies efficiently, we screened and analyzed the haplotypes that are equal or greater than 2 for the 3 samples. Repeated sequencing showed that the top 6 variants are completely accordant, only the proportions of the first 2 variants have some discrepancy, but doesn’t affect the ranking of variants, compared with higher or lower proportion than the real ratio from Sanger sequencing, which embodies superb repeatability and accuracy of high-throughput sequencing technologies in studying quasispecies. Also conventional Sanger sequencing has great randomness in the mutation rate, which may primarily be the results of too few clones selected by Sanger sequencing, when compared with deep sequencing.

The reliability of Sanger sequencing was simulated and analyzed in researching quasispecies under antibody selection pressure via 1000-time resample with replacement based on high-throughput data. 12 samples were selected with different antibody values from hundreds of samples, then the entropy values were calculated ranged from 0.15 to 0.73. When the antibody is negative, the entropy is always lower than 0.4. And the resample numbers that can truly reflect the circumstances of quasispecies had large individual variation. This may be connected with the tolerance to ALV-J for different chickens. But when the antibody selection pressure is positive, the sequence entropy is higher than 0.45, and the entropy value is positively correlated with antibody titer. The higher the entropy is, the lower proportion of dominant quasispecies occupies. The real circumstance of quasispecies can be generally reflected through at least 800 clones, which is impossible to accomplish via Sanger sequencing technologies.

In summary, the Illumina MiSeq platform is better suited for detecting rare variants whereas the Sanger has a shorter turnaround time. Also, the analysis of conventional sequencing by simulation with resampling that we propose here will also help to standardize quasispecies researching under different selection pressure based on next-generation sequencing data. Studying quasispecies accurately and comprehensively could help us to know the virus integrally and dynamically, which is beneficial to prevent, diagnose, and treat the virus infection more reasonably.

## Methods

### Sample preparation

In order to comprehensively compare the quasispecies between Sanger and MiSeq deep sequencing during the infection of ALV-J NX0101 strain, plasma samples were collected from specific-pathogen-free (SPF) chickens infected with ALV-J infectious clone rNX0101 [26]. The plasma samples with persistent viremia and antibody were determined by the IDEXX antibody kit (IDEXX, USA). In this experiment, we chose 3 samples with persistent viremia and antibody for analyzing, and 50 μl plasma was used to extract viral RNA using the MagMAX-96 viral RNA isolation kit (Ambion, Austin, TX, USA) according to the manufacturer’s instructions. The animal infection protocol was reviewed and approved by the Shandong Province Animal Ethics Committee.

### Cloning and Sanger sequencing

First, we designed a pair of primers targeting for gp85 gene from NX0101 (GenBank accession number: DQ115805).gp85-F: CCGGAGAAGACACCCTTGCT (corresponding to NX0101 position 5380–5399); gp85-R: GCAAATATCCGGGCTGTC (corresponding to NX0101 position 6453–6470). The polymerase chain reaction (PCR) conditions with high fidelity enzyme ex taq were as followed: denaturation at 95 °C for 5 min; 32 cycles of denaturation at 95 °C for 50 s, annealing at 55 °C for 40s, and extension at 72 °C for 1 min; and a final elongation step at 72 °C for 10 min. The PCR products were used for cloning by ligation with PMD18-T vector, following the manufacture’s instructions. Plasmids were chemically transformed into DH5α competent cells using heat shot method. Then smear the transformed bacteria on a Luria broth (LB) agar plate with 0.1 % ampicillin, following an incubation overnight at 37.5 °C.

In order to reduce error, we mixed all the positive clones for the 3 samples. A total of 72 positive clones were selected and sequenced using the 3730xl sequencing platform with following steps: 1) preparation of plasmid. 2) check the DNA concentration and quality. 3) PCR for sequencing. 4) purification of the PCR products. 5) denaturation. 6) computer electrophoresis. 7) data analysis.

### MiSeq sequencing

PCR primers for amplifying two highly variable regions of the gp85 gene from NX0101 strain (gp85-A including the vr2 and hr1 regions and gp85-B including the hr2 and vr3 regions) were as follows: gp85-A-F: 5′-GGCATTCCACAGTATCCTC-3′, gp85-A-R:5′-CGTCCATGATTGGTTGACA-3′;gp85-B-F:5′-GTCCAATAAACGTAGAGAG-3′,gp85-B-R:5′-GCCCTGTCCCCACAAATCA-3′. Each sample was amplified using a forward primer with a six-digit error-correcting barcode as described earlier [27]. In addition, a 2-bp GT linker was added between barcode and the 5′ end of F primer to avoid a potential match between the barcode and target sequences. The PCR conditions comprised of an initial denaturation at 94 °C for 2 min; 32 cycles of denaturation at 94 °C for 15 s, annealing at 58 °C for 30 s, and extension at 68 °C for 30 s; and a final elongation step at 68 °C for 10 min. All the PCR products were run on 1 % agarose gels and extracted using the QIAquick gel extraction kit (Qiagen, Hilden, Germany) and quantified using a spectrophotometer (NanoDropND-1000,Thermo Fisher Scientific, Waltham, MA). The barcode-tagged PCR products were pooled with the other samples. Samples were purified using the QIAquick PCR Purification Kit (Qiagen, Hilden, Germany). The DNA was end-repaired, A-tailed and PE-adapter ligated using the Paired-end Library Preparation Kit (Illumina, San Diego, CA, USA). The PCR product was gel purified and sequenced using MiSeq PE250 at the Beijing Genomics Institute (Shenzhen, China) according to the manufacturer’s instructions. A base-calling pipeline (Sequencing Control Software, SCS; Illumina) was used to process the raw fluorescent images and the call sequences.

### Data filtration and analysis

According to the in-house procedures, reads of low quality were pre-processed removed. If two paired-end reads overlapped, the consensus sequence would generate using COPE. V1.2.1 [28]. Reads were filtered using instrument quality scores and aligned to the reference ALV-J sequence (DQ115805) using a codon-aware version of the Smith–Waterman algorithm. Multiple sequences alignment were performed using Muscle [29] and then manually adjusted.

In order to investigate quasispecies diversity, we calculated the normalized entropy using clean reads of each sample with the formula followed [30]:

$$ {S}_n=-{\displaystyle {\sum}_{i=1}^n{p}_i \ln \left({p}_i\right)}/ \ln (h), $$

pi indicated the proportion of the ith haplotype from total reads; h is the total number of the reads. With the aid of re-sampling strategy with replacement from high-throughput sequencing data, 12 samples with different entropy were chose, the influence on final results has been determined by simulating a different quantity of sequences from Sanger sequencing. Specific method is as follows: In the range of preset (N, M), re-sampling numbers were set according to pretest. Each sample choose and calculated n sequences from re-sampling with replacement for 1000 times repeat (*n =* 10, 30, 50, 100, 200, 300, 500, 1000). Then analyze the results of n sequences from 1000-times re-samplings as followed:

1. How many times the haplotype ranked first in re-sampling are the same with the number one haplotype in high throughput sequencing.
2. The average proportion and variance of the haplotype identical with the haplotype ranked first in high throughput sequencing.
3. The average proportion and variance of the haplotype identical with the haplotype ranked second in high throughput sequencing.
4. The average normalized entropy and variance of the n sequences.

The confidence interval of high-throughput sequencing entropy using 1000 times bootsrap estimation. The mutational frequency of sequence data were plotted by Weblogo. While scatter diagrams were analyzed with OriginPro 8.
